# Vitamin D in 2020: An Old Pro-Hormone with Potential Effects beyond Mineral Metabolism

**DOI:** 10.3390/nu12113378

**Published:** 2020-11-03

**Authors:** Marie Courbebaisse, Etienne Cavalier

**Affiliations:** 1 Faculty of Medicine of Paris Descartes, Paris University, 75006 Paris, France; 2 Physiology Department, European Georges-Pompidou Hospital, AP-HP, 75015 Paris, France; 3 INSERM U1151, 75015 Paris, France; 4 Department of Clinical Chemistry, University of Liège, B-4000 Liège, Belgium; etienne.cavalier@chuliege.be

Vitamin D is not a vitamin but a pro-hormone. It can be found as two forms, either vitamin D_2,_ which is the form found in plants, and vitamin D_3_, which can be found in food (mainly fatty fish) but which is also produced by the cutaneous conversion of 7-dehydrocholesterol under the action of UVB. In human diets, vitamin D_2_ is rarely found and vitamin D_3_ represents the major part of vitamin D intakes. Both forms can also be found as supplements. Vitamin D, either from food, supplements or produced by the skin undergoes a first hydroxylation in the liver on carbon 25 under the effect of CYP2R1, a 25-hydroxylase, leading to the production of 25-hydroxy vitamin D (25OHD). 25OHD then undergoes a second hydroxylation on carbon 1 under the effect of a 1α-hydroxylase (CYP27B1) expressed in the proximal tubular cells of the kidney and is thus converted into 1,25 dihydroxyvitamin D or calcitriol, the active form of vitamin D, a steroid hormone with actions on mineral metabolism [1]. 25OHD is also a substrate for the 1α-hydroxylase expressed in several extra-renal tissues. These extra-renal tissues are dependent on adequate levels of 25OHD to ensure adequate local calcitriol production, which is responsible for the extra skeletal autocrine and paracrine actions of vitamin D, such as the modulation of renin and insulin synthesis, regulation of cell proliferation and apoptosis, and of innate and adaptative immunity [2]. 

The assessment of vitamin D status is based on the measurement of the serum concentration of 25OHD. Although there is a consensus to define severe vitamin D deficiency as a serum 25OHD concentration below 12 ng/mL (30 nmol/L), the definition of vitamin D adequate level is less consensual. Whereas the US Institute of Medicine considers that serum 25OHD concentrations between 20 and 50 ng/mL (50–125 nmol/L) [3] are adequate for the general population, the Endocrine Society considers 25OHD levels below 30 ng/mL (75 nmol/L) as insufficient [4], at least in some groups of patients (patients with osteoporosis or at risk of osteoporosis, patients with chronic kidney disease, patients with intestinal malabsorption and elderly at high risk of falling). 

In addition to its protective effect against rickets and osteomalacia, vitamin D sufficiency has been associated with a reduced risk of many diseases, including type 2 diabetes mellitus, major cardiovascular events, cancers, infectious diseases and chronic kidney disease [5]. Of note, high 25OHD concentrations (above 40 ng/mL) have also been associated with an increased risk of cardiovascular events [6,7], potentially attributable to the increase in vascular and valvular calcifications. Randomized trials and meta-analyzes of randomized trials show that vitamin D supplementation reduces total fractures in elderly people, falls in frail elderly people, respiratory infections in the general population, blood pressure in hypertensive patients with vitamin D deficiency, and all causes of death [5]. 

However, the results of two recent large-scale US randomized controlled trials showed no effect of high doses of vitamin D on cardiovascular events and cancer incidence in the general population [8] or on diabetes incidence among persons at high risk of type 2 diabetes [9]. 

How should we thus position ourselves in 2020? Should we say “*stop*” or “*yet*”? Indeed, the aging of the population, but also new challenges and discoveries, are still triggering our interest for vitamin D. In this Special Issue of Nutrients entitled “Vitamin D in 2020: Stop or yet?”, international experts, researchers and authors have been invited to submit their latest research ranging from epidemiological studies to interventional trials. 

This Special Issue presents a compendium of excellent observational and interventional clinical studies regarding vitamin D. The high frequency of vitamin D insufficiency or deficiency among adults and the elderly [10], but also among mothers and their infants and in breastmilk [11], are underlined. Although vitamin D_3_ production decreases with age, the significant role of outdoor sun exposure to increase serum vitamin D_3_ concentrations in vivo in younger and older adults is demonstrated [12], and the barriers towards sun exposure and the potential improvement strategies to promote safe sun exposure to produce an optimal level of vitamin D are discussed [13]. Regarding 25OHD assessment, it is specified that the detection of 3-Epi25(OH)D_3_ using liquid chromatography-tandem mass spectrometry prevents the overestimation of 25OHD and misclassification of vitamin D status [11]. Interventional studies showed no effect of cholecalciferol on body weight reduction in obese children participating in a weight management program [14], or on bone mineral density in children with hypercalciuria [15]. This last study emphasizes the safety of vitamin D treatment (400–800 IU/day) on calciuria and on the evolution of stones in the urinary tract in these children [15]. In post-menopausal women, ergocalciferol 40,000 IU/week did not change vulvovaginal atrophy compared to placebo, but improved vaginal pH and visual analog scale of vulvovaginal atrophy symptoms between baseline and 12 weeks in the vitamin D group [16]. In very old adults, 25OHD concentrations below 25 nmol/L were associated with moderate and severe disability trajectories, even after adjustment for sex, living in an institution, season, cognitive status, body mass index and vitamin D supplementation [17]. Of note, this association disappeared after further adjustment for physical activity [17]. Interestingly, vitamin D receptor polymorphisms are reported to influence individual susceptibility to develop chronic autoimmune liver disease and affect quality of life of these patients [18]. Finally, an in vitro study shows that treatment of head and neck cancer cell lines with vitamin D alters multiple cancer pathways at genes and proteins levels, supporting a potential role for vitamin D in cancer inhibition [19]. 

In conclusion, this Special Issue reinforces the high prevalence of vitamin D deficiency and insufficiency in the general population and the safety of this low cost molecule and open new perspectives regarding extra skeletal effects of vitamin D. We thank all the authors for their contributions to this Special Issue dedicated to an old pro-hormone whose potential interests beyond mineral metabolism are still being investigated. 
